# Combination Approaches with Immune-Checkpoint Blockade in Cancer Therapy

**DOI:** 10.3389/fonc.2016.00233

**Published:** 2016-11-01

**Authors:** Maarten Swart, Inge Verbrugge, Joost B. Beltman

**Affiliations:** ^1^Division of Toxicology, Leiden Academic Centre for Drug Research, Leiden University, Leiden, Netherlands; ^2^Division of Immunology, Netherlands Cancer Institute, Amsterdam, Netherlands

**Keywords:** cancer immunotherapy, checkpoint blockade, CTLA-4, PD-1

## Abstract

In healthy individuals, immune-checkpoint molecules prevent autoimmune responses and limit immune cell-mediated tissue damage. Tumors frequently exploit these molecules to evade eradication by the immune system. Over the past years, immune-checkpoint blockade of cytotoxic T lymphocyte antigen-4 and programed death-1 emerged as promising strategies to activate antitumor cytotoxic T cell responses. Although complete regression and long-term survival is achieved in some patients, not all patients respond. This review describes promising, novel combination approaches involving immune-checkpoint blockade in the context of the cancer-immunity cycle, aimed at increasing response rates to the single treatments. Specifically, we discuss combinations that promote antigen release and presentation, that further amplify T cell activation, that inhibit trafficking of regulatory T cells or MSDCs, that stimulate intratumoral T cell infiltration, that increase cancer recognition by T cells, and that stimulate tumor killing.

## Introduction

**Figure 1:**
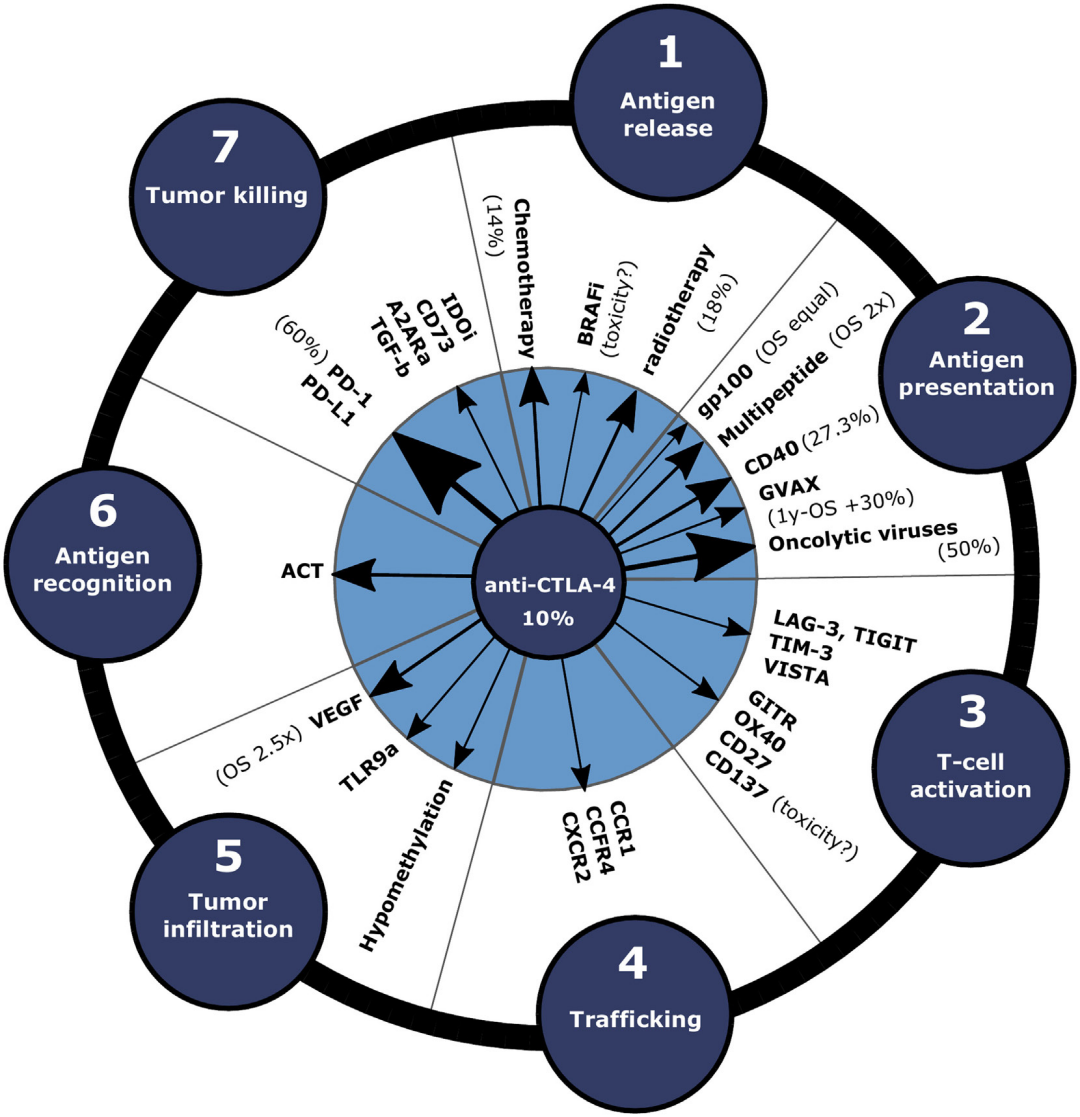
**Clinical efficacy of combination approaches with anti-CTLA-4 depicted in the cancer-immunity cycle**. In order to enhance the response rate of immune-checkpoint blockade, anti-CTLA-4 can be combined with therapies that promote various steps of the cancer-immunity cycle. Arrow size indicates the efficacy increase compared to anti-CTLA-4 monotherapy in clinical and preclinical studies. Clinical response rates or OS data compared to anti-CTLA-4 only for the treatment of melanoma patients are annotated between brackets. Abbreviations: A2ARa, adenosine 2a receptor antagonist; ACT, adoptive cell transfer; BRAFi, BRAF^V600E^ inhibitors; GVAX, cellular vaccines consisting of irradiated GMCSF-producing tumor cells; IDOi, IDO inhibitor; MEKi, MEK inhibitors; OS, overall survival; TLR9a, TLR9 agonist.

Historically, the importance of the immune system in restraining cancer development has been demonstrated by the observation that immune-deficient patients and organ-transplant recipients treated with immunosuppressive drugs have an increased risk to develop cancer (1). The steps involved in the development and maintenance of tumor immunity were recently conceptualized in the “cancer-immunity” cycle (and schematically depicted in Figure 1, outer circle) (2). This cycle describes the steps that are required to generate tumor-specific cytotoxic T lymphocytes [CTLs, also known as CD8^+^ (effector) T cells] that are capable of killing tumor cells. Defects/impediments in the cycle due to inhibitory receptor signaling frequently lead to evasion of tumor eradication by the immune system. This may explain the clinical success of immune-checkpoint therapies. Two important immune-checkpoint molecules include cytotoxic T lymphocyte antigen-4 (CTLA-4, also known as CD152) and programed death-1 (PD-1, also known as CD279).

### Cytotoxic T Lymphocyte Antigen-4

**Figure 2:**
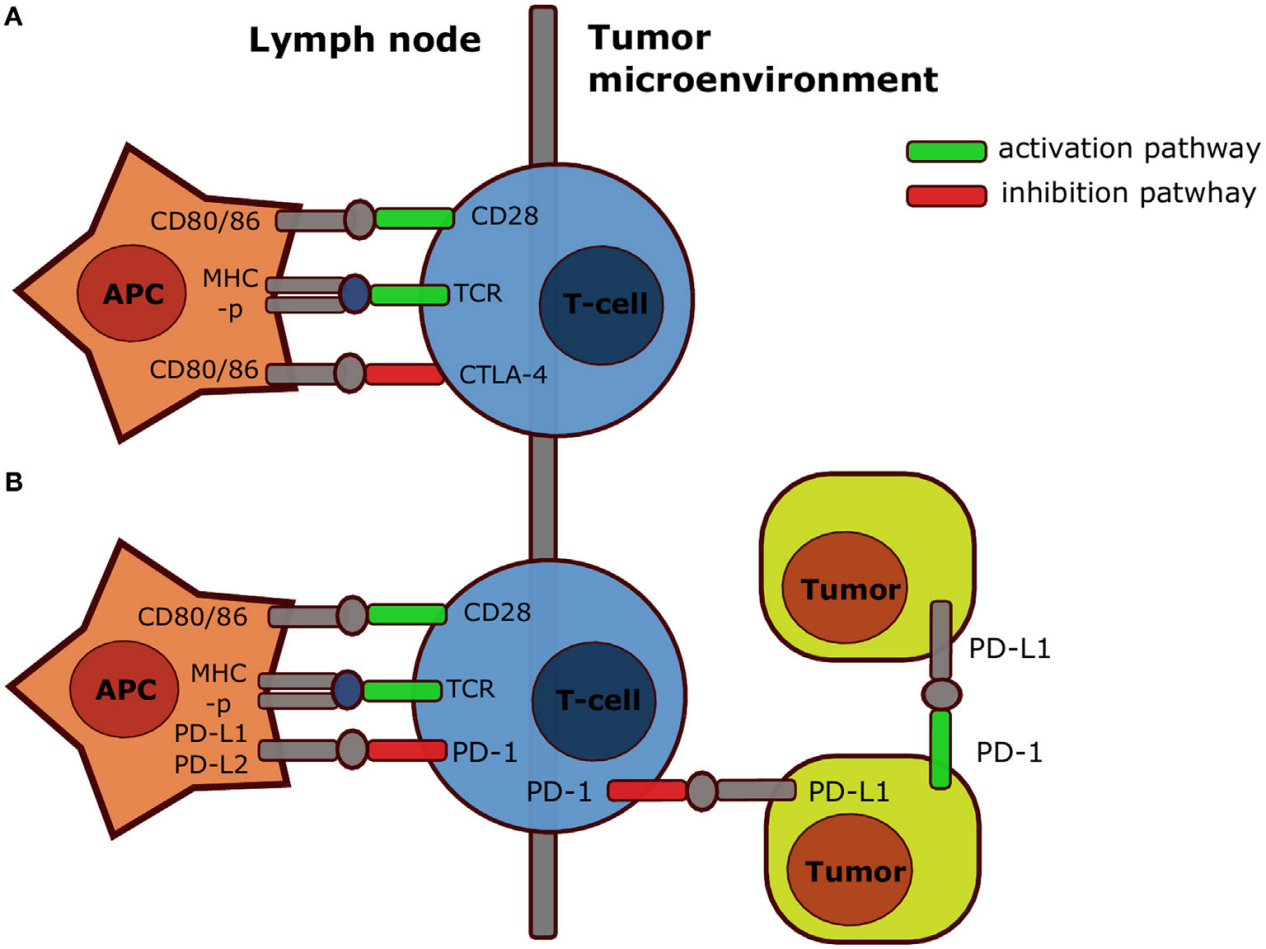
**Simplified mechanism of action CTLA-4 and PD-1**. Both CTLA-4 **(A)** and PD-1 **(B)** inhibit T cell activation in secondary organs after interaction with respectively B7 or PD-L1 and PD-L2. However, PD-1 also inhibits T cell responses after interaction with PD-L1 on tumor cells, and PD-1 expressed by a specific subset of tumor cells also contributes directly to tumorigenesis upon interaction with PD-L1 on tumor cells or stroma cells. Abbreviations: APC, antigen-presenting cell; CD, cluster of differentiation; CTLA-4, cytotoxic T lymphocyte antigen-4; MHC-p, peptide-major-histocompatibility complex; PD-1, programed death-1; PD-L1, programed death-ligand-1; TCR, T cell receptor. Note that the scheme is highly simplified: in reality CTLA-4 and PD-1 act through multiple mechanisms.

Like CD28, CTLA-4 binds to the co-stimulatory molecules CD80 or CD86, but instead attenuates T cell activation (3). Therefore, CTLA-4 mainly exerts its immunosuppressive effect during activation in secondary lymphoid organs (Figure 2A) (4, 5). However, CTLA-4 signaling also enhances immunosuppression in other ways, e.g., by promoting the activity of regulatory T cells (Tregs) inside the tumor-microenvironment (6, 7), and the relative importance of these different mechanisms is not fully understood nor their contribution to therapy success. The anti-CTLA-4 monoclonal antibody ipilimumab (fully human IgG1, Bristol-Myers Squibb) increased the median overall survival (OS) among metastatic melanoma patients in a pooled analysis of phase II and III data and some patients even survived 10 years after treatment (8). However, 60% of the patients treated with anti-CTLA-4 experienced immune-related adverse events, which were severe (grade 3 or 4) in 10–15% of the patients (9).

Anti-CTLA-4 treatment has also been studied in non-melanoma tumors, demonstrating an 8% partial response rate in patients with metastatic renal cell carcinoma (RCC) in a phase II study (10). Nevertheless, anti-CTLA-4 treatment is generally not effective in non-melanoma tumors, which might for instance be due to a low fraction of tumor-reactive T cells in these tumors, high expression of PD-L1, or the secretion of anti-inflammatory cytokines. Overall, long-term survival can be obtained with anti-CTLA-4 treatment in melanoma patients, but the toxicity is quite high and it is typically less effective in other tumor types.

### Programed Death-1

Similar to CTLA-4, PD-1 signaling within secondary lymphoid organs impairs T cell priming and enhances differentiation into Tregs (11, 12). Moreover, because PD-L1 expression is frequently upregulated in cancers and tumor-infiltrating lymphocytes (TILs) can express high levels of PD-1 (4, 13), PD-1 inhibits T cell responses in the tumor. PD-1 can also be expressed on tumor cells, such as melanoma subpopulations, and contribute to tumorigenesis (14). Therefore, a part of the efficacy of anti-PD-1 therapy may be due to inhibition of this cell-intrinsic pathway active in some tumor cells (Figure 2B) (14). As expected from the predominantly peripheral role of PD-1, *Pdcd1^−/−^* (the gene encoding for PD-1) mice developed mild, organ-specific autoimmune responses (15, 16). Large phase I studies led to the prompt approval of the anti-PD-1 monoclonal antibodies pembrolizumab (humanized IgG4, Merck) and nivolumab (fully human IgG4, Bristol-Myers Squibb, Ono Pharmaceuticals) for patients with unresectable or metastatic melanoma not responding to anti-CTLA-4 (17–19). Importantly, anti-PD-1 was superior to anti-CTLA-4 in the treatment of advanced melanoma in terms of progression-free survival (PFS; 47.3 versus 26.5%) (20). Because severe (grade 3–5) side effects also occurred less frequently in anti-PD-1-treated (13.3%) compared with anti-CTLA-4-treated patients (19.9%), anti-PD-1 treatment is currently the first-line treatment for unresectable or metastatic melanoma in the USA and the EU. In addition, the FDA approved anti-PD-1 for the treatment of Hodgkin lymphoma, non-small-cell lung carcinoma (NSCLC), RCC, and head and neck squamous cell carcinoma (HNSCC), because clinical trials demonstrated the safety and efficacy in these cancer types (21–26). Anti-PD-1 might also improve the treatment of bladder, gastric, ovarian, and triple negative breast cancer (4, 19). Furthermore, anti-PD-L1 (atezolizumab) was recently approved for the treatment of bladder cancer (urothelial carcinoma) (27). In summary, anti-PD-1 is less toxic yet more effective than anti-CTLA-4 and is also effective in the treatment of non-melanoma tumors.

## Improving Tumor Regression upon CTLA-4 or PD-1 Blockade

Despite the general success of checkpoint therapies, not all patients respond or achieve only partial tumor regression to anti-PD-1 or anti-CTLA-4 monotherapy (20). This is probably due to impediments somewhere in the cancer-immunity cycle (Figure 1): release of cancer antigens (step 1), antigen presentation (step 2), T cell priming and activation (step 3), T cell trafficking to tumors [step 4; note that, in this review, we specifically consider blocking the trafficking of immunosuppressive Tregs and myeloid-derived suppressor cells (MDSCs)], T cell infiltration into the tumor (step 5), cancer cell recognition by T cells (step 6), and killing of tumor cells (step 7). Therefore, higher response rates may be achieved using combination approaches of anti-PD-1 or anti-CTLA-4 with therapies that stimulate various steps of the cancer-immunity cycle, which we will discuss in this review. In brief, this involves combinations with conventional (e.g., chemotherapy and radiotherapy) and targeted therapies to promote antigen release (step 1) (28); combinations with vaccination to promote antigen presentation (step 2); combinations with agonists for co-stimulatory molecules or blockade of co-inhibitory molecules to further amplify T cell activation (step 3); combinations with trafficking inhibition of Tregs or MSDCs (step 4); combinations with anti-vascular endothelial growth factor (VEGF) to stimulate intratumoral T cell infiltration (step 5); combinations with adoptive cell transfer (ACT) to increase cancer recognition by T cells (step 6); and combinations that stimulate tumor killing (step 7). Finally, individualized treatment, based on biomarkers that predict clinical responses, could potentially optimize the management of various cancer types (29). In the following, we will discuss the progress with respect to the mentioned combination strategies step by step.

## Combinations with Stimulation of Antigen Release and Danger Signals (Step 1)

Chemotherapy, targeted therapies, and radiotherapy can promote immunogenic cell death (ICD) of tumor cells. ICD results in the release of tumor antigens and “danger signals,” also known as damage-associated molecular patterns (DAMPS), such as calreticulin, ATP, type I IFN, and non-histone chromatin-binding protein high-mobility group box 1 (HMGB1) (30, 31). Binding to their receptors (CD91, the purinergic receptors P2RX7 and P2RY2, IFNAR, and the toll-like receptor TLR4, respectively) on DCs, results in their activation, enhanced antigen presentation, upregulation of co-stimulatory receptors, and induction of adaptive immune responses (32), whereas cell death that is “immunologically silent” induces tolerance.

### Chemotherapy

Promising preclinical studies have shown that chemotherapy can indeed sensitize tumors to immune-checkpoint blockade by promoting T cell activation and infiltration into the tumor (33). Moreover, chemotherapy, such as by cisplatin, can also enhance responses to T cell based immune therapies by sensitizing the tumor cells to T cell-induced death rather than by ICD (34). For example, cisplatin has been shown to synergize with synthetic long peptide (SLP) vaccination and improve tumor-cell killing in a preclinical tumor model (35). With respect to the clinical application, chemotherapy (dacarbazine) combined with anti-CTLA-4 (ipilimumab) was first tested in metastatic melanoma patients. A phase II study showed that more patients responded to dacarbazine plus anti-CTLA-4 when compared to anti-CTLA-4 alone (14.3 versus 5.4%) (36). In addition, a phase III study demonstrated that this combination slightly increased the OS, when compared to dacarbazine alone (11.2 versus 9.1 months) (37) (note the difference between the latter two studies in terms of the monotherapy). However, immune-related severe adverse events (grade 3 or 4) also increased (56.3 versus 27.5%) (37).

Since chemotherapeutics promote antigen release and may promote the activation of tumor-specific T cells, combined chemotherapy and immune-checkpoint blockade may also be clinically effective in non-melanoma tumors. However, chemotherapy (paclitaxel and carboplatin) followed by anti-CTLA-4 (ipilimumab) resulted in only a minor increase of the immune-related PFS in a phase II and phase IIIb/IV study in NSCLC and extensive disease SCLC patients, compared with chemotherapy alone (38, 39). In conclusion, despite promising preclinical studies, combination therapies of immune-checkpoint blockade with chemotherapy have so far only slightly enhanced the clinical efficacy, while amplifying adverse effects. The lack of efficacy might be attributed to limited induction of ICD by chemotherapy. As ATP is thought to be important in the recruitment and activation of DCs during ICD, combinations of chemotherapy and inhibition of the ATP degrading enzyme CD39 followed by immune-checkpoint blockade might improve the clinical efficacy (40, 41).

### Targeted Therapies

Targeted therapies can also restore T cell activation, but subsequent expression of PD-L1 on tumor cells can lead to immune escape. For example, clinical data demonstrated that such escape occurred within 10–14 days after BRAF inhibition in tumors of patients with metastatic melanoma (42). Thus, combination approaches with immune-checkpoint blockade may function synergistically. However, a phase I trial combining BRAF^V600E^ inhibitor vemurafenib with anti-CTLA-4 (ipilimumab) in metastatic melanoma was halted due to severe hepatotoxicity (43). To date, such hepatoxity did not occur in an ongoing phase I trial with the BRAF^V600E^ inhibitor dabrafenib in combination with anti-CTLA-4 (ipilimumab) (44, 45), but clinical efficacy data are not yet available. Because PD-L1 is upregulated on tumor cells following BRAF inhibition, and anti-PD-1 therapy is less toxic compared to anti-CTLA-4, combining BRAF^V600E^ inhibitors with anti-PD-1 therapy seems promising. Indeed, preclinical data showed that the combination of anti-PD-1 or anti-PD-L1 with BRAF inhibition enhanced antitumor responses in a BRAF/PTEN inducible melanoma model (46), thus warranting clinical studies. Still, BRAF^V600E^ inhibition can potentially lead to activation of MAPK signaling in T cells, since wild-type BRAF is not inhibited. This may result in exhaustion or enhance immune suppression (47).

Until recently, the preclinical and clinical assessment of MEK inhibitors (e.g., tramatinib) in combination with immune-checkpoint blockade was limited, because MEK inhibitors disrupt T cell function transiently *in vitro* (48). Nevertheless, a synergistic effect of trametinib plus anti-PD-1, anti-PD-L1, or anti-CTLA-4 was observed in the preclinical CT26 carcinoma model (49). Triple therapy with MEK inhibitors, BRAF^V600E^ inhibitors, and anti-CTLA-4 may potentially optimize antitumor T cell responses with limited autoimmune side effects, since the additional MEK inhibitors might circumvent T cell hyper-activation caused by BRAF inhibition (47). Nevertheless, the combination of MEK inhibitors (trametinib), BRAF^V600E^ inhibitors (dabrafenib), and anti-CTLA-4 (ipilimumab) resulted in severe gastrointestinal toxicity in patients with metastatic melanoma in a phase I/II study (45). In contrast, MEK inhibitors (trametinib) and BRAF^V600E^ inhibitors (dabrafenib) have been successfully combined with anti-PD-L1 (durvalumab; MEDI4736) in melanoma patients in a phase I study (50). Thus, MEK or BRAF^V600E^ inhibitors might be safely and effectively used in combination with anti-PD-L1, but little clinical efficacy data are currently available.

### Radiotherapy

Radiotherapy induces local tumor cell death, but can also modulate local and systemic immunity, in essence acting as an *in situ* vaccine. Radiotherapy can promote CD8^+^ T cell responses to radiotherapy-induced peptides (51), natural (52, 53) or exogenous tumor antigens (54, 55), or by promoting immune control within the tumor-microenvironment (51, 56–58) [reviewed in Ref. (59)]. CD8^+^ T cells can contribute to radiotherapy-induced reduction of the primary tumor (55), and radiotherapy can even induce regression of lesions that reside outside the field of radiotherapy, called the “abscopal effect.” However, this is a very rare phenomenon, reported 34 times since 1969 [reviewed in Ref. (60)], indicating that radiotherapy alone sub-optimally engages the cancer-immunity cycle (or does not overrule all the impediments that may still be there).

However, local and systemic responses may be achieved at the same time when radiotherapy is combined rationally with immunotherapy. Indeed, the Demaria/Formenti groups first demonstrated that radiotherapy combined with anti-CTLA-4 improved control of irradiated tumors and reduced non-irradiated lung metastases in preclinical models (61, 62). Similarly, PD-L1 or PD-1 blockade and radiotherapy-induced local combined responses in mouse models for colon carcinoma (MC38) and mammary tumors (4T1 and TUBO) (63), and resulted in long-term survival in a mouse model for intracranial gliomas (GL261) (64). In the latter case, long-term immunologic memory was achieved, because rechallenged, long-term surviving mice did not develop new tumors.

A number of clinical case studies showed that concurrent treatment with radiotherapy and anti-CTLA-4 (ipilimumab) promoted an abscopal effect in metastatic melanoma (3 × 9.5 Gy) as well as in NSCLC (5 × 6 Gy in 10 days) (65, 66). However, it is unclear whether this effect is caused by the combination of radiotherapy and anti-CTLA-4 (systemic effect of combination) or anti-CTLA-4 alone (systemic effect of immunotherapy), since the latter control was obviously not included in these case studies.

Confirming the challenge of obtaining systemic combined effects between radiotherapy and aCTLA-4 comes from a phase I/II study with castration-resistant prostate cancer (CRPC) patients, in which the tumors were first irradiated (1 × 8 Gy), followed by anti-CTLA-4 (ipilimumab) administration 2 days later in an attempt to maximize antigen presentation (67). In this study, relatively few severe adverse events (10%) were reported and a complete response occurred. However, no differences in median survival were found in a phase III trial among metastatic CRPC patients applying radiotherapy plus anti-CTLA-4 (ipilimumab) or placebo (11.2 versus 10.0 months) (68), again indicating that systemic combined effects between radiotherapy and anti-CTLA-4 are sub-optimal. Finally, a recent phase I study in metastatic melanoma patients reported an 18% abscopal response rate, but this is also expected from anti-CTLA-4 treatment alone (69). Anti-CTLA-4 treatment can result in the upregulation of tumoral PD-L1 expression (69). Indeed, the addition of anti-PD-1 or anti-PD-L1 to radiotherapy plus anti-CTLA-4 in a mouse model increased local combined responses and achieved cures in up to 80% of the animals, but combined effects on non-irradiated tumors were not assessed in this setting (69).

Hence, high (local) response rates are currently only achieved in combination with double immune-checkpoint blockade, and convincing clinical abscopal effects with radiotherapy and immune-checkpoint blockade are yet to be reported. In order to optimize the efficacy of combination approaches with radiotherapy, it is important to mechanistically understand how radiotherapy can optimally induce ICD to kickstart a new T cell response, which likely depends on the tumor type and location (hypoxic versus normoxic environment) and how irradiated and distal tumor-microenvironment are influenced by radiotherapy. Then, irradiation dose, scheduling, size of radiotherapy field (note: want this as small as possible to prevent damage to circulating lymphocytes that circulate through the RT field), timing, and delivery method can be optimized to achieve local and systemic therapeutic effects with immune-checkpoint inhibitors (70–72). For more details on preclinical and clinical efficacy of radiotherapy combined with aCTLA-4 see an elegant, recent review by Vanpouille-Box et al. (73).

In summary, the preclinical studies on combination approaches of immune-checkpoint blockade with stimulation of antigen release are promising, yet the clinical efficacy is currently limited. Furthermore, combination therapies with chemotherapy of BRAF-targeted therapies are potentially accompanied by severe side effects. Combination approaches with radiotherapy seem to have a more favorable toxicity profile, but, to date, local and abscopal antitumor responses are limited.

## Combinations with Stimulation of Antigen Presentation and Co-Stimulation (Step 2)

Peptide vaccines, live attenuated vaccines, cellular vaccines, oncolytic viruses, and agonists for co-stimulatory molecules on antigen-presenting cells (APCs), such as DCs, can be used to stimulate antigen presentation.

### Peptide

In a phase I study with advanced melanoma patients, vaccination with a modified sequence from glycoprotein 100 (gp100), a melanoma tumor antigen, increased the frequency of melanoma-specific CD8^+^ T cells (74). Nevertheless, the tumors progressed, implying that vaccination may benefit from combination with immune-checkpoint blockade. However, anti-CTLA-4 (ipilimumab) plus gp100 peptide vaccine did not improve the OS, compared with anti-CTLA-4 alone (10.0 versus 10.1 months) in a phase III trial in advanced melanoma patients (9). Thus, multiple antigens may need to be targeted to induce sufficient antitumor CD8^+^ T cell responses. Such a multi-peptide vaccine (gp100, MART-1, NY-ESO-1) increased the number of gp100^+^, MART-1^+^, and NY-ESO-1^+^ CD8^+^ T cells in advanced melanoma patients when combined with anti-PD-1 (nivolumab), and a promising 1-year survival of 87% was obtained in this phase I trial (75). Thus, early clinical data indicate that multi-peptide vaccines combined with immune-checkpoint blockade are highly promising.

### Live Attenuated Vaccines

Vaccination strategies in combination with immune-checkpoint blockade are also studied in non-melanoma tumors. Currently, a vaccine with a live-attenuated *Listeria* strain encoding the human papillomavirus (HPV) 16 oncoprotein E7 (ADXS11-001) in combination with anti-PD-L1 (durvalumab) is studied in a phase I/II trial in patients with cervical cancer or HPV-positive head and neck cancer (NCT02291055). Furthermore, a live-attenuated *Listeria* strain encoding for prostate-specific antigen level (PSA) (ADXS31-142) in combination with anti-PD-1 (pembrolizumab) is studied in a phase I/II trial in patients with prostate cancer (NCT02325557). In summary, combination approaches with live attenuated viruses are currently tested in the clinic, but clinical safety and efficacy data are not available yet.

### Cellular Vaccines

Clinical trials combining checkpoint therapy with cellular vaccines are also ongoing, such as a phase I trial combining MART-1_26–35_-loaded DC injection with anti-CTLA-4 (tremelimumab) in metastatic melanoma patients (76) and with anti-PD-1 (nivolumab) in patients with recurrent brain tumors (NCT02529072).

However, cellular vaccines consisting of irradiated GM-CSF-producing tumor cells (GVAX) may be more suitable, since they deliver multiple antigens. Granulocyte-macrophage colony-stimulating factor (GM-CSF) attracts DCs, stimulates the differentiation of monocytes into DCs, and amplifies DC cell maturation and antigen presentation (77, 78). Preclinical studies showed that subcutaneous injection of GVAX plus CTLA-4 blockade synergistically promoted tumor eradication in the B16 mouse melanoma model (response rate 80 versus 16% with GVAX alone) (79) due to an increase in CD8^+^ T cells and in effector T cell/Treg ratio in the tumor (80). GVAX plus PD-1 blockade also enhanced survival in the murine B16 melanoma, CT26 colon carcinoma, and pancreatic ductal adenocarcinoma models (81, 82). Furthermore, GVAX plus double checkpoint blockade (anti-CTLA-4 and anti-PD-1) enhanced tumor rejection to 100% in CT26 colon carcinoma and to 75% in ID8 ovarian carcinoma, compared to, respectively, 75 and 50% with double checkpoint blockade only (83). It should be noted that depigmentation was observed in 56% of the mice after combined GVAX and immune-checkpoint blockade (79), which correlated with antitumor activity. Clinical studies with cellular vaccines and checkpoint therapy are still rare. In a phase I/II trial with metastatic CRPC patients, treatment with GVAX plus anti-CTLA-4 (ipilimumab) was found to be safe and decreased the PSA by ≥50% in 25% of the patients (84). Thus, preclinical and early clinical data of cellular vaccines in combination with immune-checkpoint blockade are promising, warranting future clinical studies.

### GM-CSF and Oncolytic Viruses

Granulocyte-macrophage colony-stimulating factor can also be directly administered instead of through production by irradiated tumor cells. In a phase II trial of systemic administration of recombinant GM-CSF (sargramostim) plus ipilimumab in metastatic melanoma patients, their 1-year survival increased upon combination therapy compared with anti-CTLA-4 alone (68.9 versus 52.9%) without toxicity differences (85). In addition, to minimize toxicity and optimize efficacy, GM-CSF can also be locally delivered in the tumor using modified oncolytic herpes viruses (talimogene laherperepvec; T-VEC) to produce GM-CSF. Such oncolytic viruses infect melanoma cells and then directly promote cell death with antigen release, stimulating antitumor immune responses (86). Intratumoral injection of T-VEC in melanoma patients in combination with ipilimumab treatment resulted in a response rate of 50% with a tolerable safety profile in a phase Ib trail. Currently, T-VEC combined with anti-CTLA-4 (ipilimumab) is studied in a phase II trial (NCT01740297) and combined with anti-PD-1 (pembrolizumab) in a phase Ib/III trail (NCT02263508), both in melanoma patients. The latter provided a clinical benefit and a phase III trial is planned (87, 88). Based on the clinical response rate of T-VEC and anti-CTLA-4, immune-checkpoint blockade with oncolytic viruses might be one of the most promising combination approaches.

### Anti-CD40 and Anti-CD47

CD40 is constitutively expressed on APCs and, like GM-CSF, agonistic antibodies for CD40 (anti-CD40) provoke DC maturation and the expression of co-stimulatory molecules. A 27.3% response rate was obtained with anti-CD40 (CP-870893) plus anti-CTLA-4 (tremelimumab) in a phase I study in melanoma patients (NCT01103635) (89). As anti-CD40 has been shown to upregulate PD-L1 in a mouse model (90), higher response rates might be obtained with anti-CD40 plus anti-PD-1. Indeed, anti-CD40 (APX005M) is currently studied in combination with anti-PD-1 (pembrolizumab) in a phase I/II trial in melanoma patients (NCT02706353). Furthermore, combination therapies with antagonistic antibodies for CD47, which provide “do not eat me” signals to APCs, might be used in the future in order to stimulate the engulfment of antigens (91). Thus, combination approaches with anti-CD40 or anti-CD47 can also be used to improve antigen presentation, but the available data show a smaller effect than other approaches to affect antigen presentation.

Overall, both the preclinical and initial clinical data for combination therapy with immune-checkpoint blockade and multi-peptide vaccines and oncolytic viruses are promising, whereas the combination with single peptide vaccines and anti-CD40 so far seem less effective.

## Combinations with Stimulation of T Cell Activation (Step 3)

Combination approaches of anti-CTLA-4 or anti-PD-1 with the blockade of other immune-checkpoints or with activation of co-stimulatory molecules may also further amplify antitumor immune responses.

### Double Immune-Checkpoint Blockade

#### CTLA-4 plus PD-1 Blockade

Preclinical models revealed that blocking of CTLA-4 or PD-1 alone led to upregulation of the unblocked pathway (92); hence, the efficacy of either monotherapy is limited by increased suppression of T cell responses through the other of the two pathways. Indeed, combined blockade of CTLA-4 and PD-1 prolonged survival in a B16 melanoma model (92), and combined PD-L1 and CTLA-4 blockade prolonged disease-free survival in the K7M2 model for metastatic osteosarcoma (93). This was due to enhanced effector T cell infiltration, proliferation, inflammatory cytokine production (e.g., IFN-γ), and an increase in the ratio of CD8^+^ T cells to Tregs and MDSCs in the tumor (92, 94).

Phase II clinical trials involving combination treatment with anti-CTLA-4 (ipilimumab) and anti-PD-1 (nivolumab) resulted in a higher response rate (61 versus 11% with anti-CTLA-4 only), more complete responses (22 versus 0% with anti-CTLA-4 only), and higher PFS in a phase III trial (11.2; anti-CTLA-4 2.9; anti-PD-1 6.9 months) (95, 96). However, severe (grade 3 or 4) treatment-related autoimmune adverse events were also amplified (55%; anti-CTLA-4 27.3%; anti-PD-1 16.3%) (96). In 2015, these studies led to the accelerated approval of anti-CTLA-4 (ipilimumab) in combination with anti-PD-1 (nivolumab) for the treatment of unresectable or metastatic melanoma in the USA. Still, optimization of this combination therapy, other combination approaches, or an individualized treatment is required to obtain clinical responses in a fraction of the patients above the current 60%. Currently, a phase I/II trial combining anti-CTLA-4 and anti-PD-1 therapy is ongoing in patients with solid tumors or sarcomas (NCT02304458), which will provide information about the efficacy and safety of double immune-checkpoint blockade in tumor types other than melanoma. Overall, double immune-checkpoint blockade with anti-CTLA-4 anti-PD-1 is currently the most effective combination approach and is approved for the treatment of melanoma.

#### Novel Immune Checkpoints

The immune-checkpoint molecules lymphocyte-activation gene-3 (LAG-3), T cell ITIM domain (TIGIT), or T cell immunoglobulin and mucin domain-3 (TIM-3) may also contribute to the immune evasion of tumor cells because they are primarily expressed on exhausted T cells (97–100). Various combinations have been tested in mouse models (Table 1). Blocking PD-1 and LAG-3 is particularly interesting because both are mainly expressed on TILs (101), which may result in a favorable safety profile due to restriction of T cell responses to the tumor-microenvironment. Results for this combination were promising in fibrosarcoma and colon cancer models, but tumor growth in the B16 melanoma model was not affected (101), possibly due to low LAG-3 and PD-1 expression on TILs in the B16 model. These observations highlight the importance to establish biomarkers that predict treatment responses to these combination approaches.

**Table 1:** **Preclinical studies double or triple immune-checkpoint blockade**.

Combination		Tumor model	Response monotherapy	Response combination therapy	Reference
Anti-CTLA-4	IDOi (1MT)	B16F10	20 and 0% OS	55% OS	(102)
		B16.SIY	0 and 0% CR	18.8% CR	(103)
Anti-PD-1 (or anti-PD-L1)	IDOi (1MT)	B16.SIY	0 and 0% CR	13.3% CR	(103)
	Anti-LAG-3	As1N	20 and 10% CR	70% CR	(101)
		B16	0 and 0% CR	0% CR	(101)
		MC38	40 and 0% CR	80% CR	(101)
	Anti-TIGIT	CT26	0 and 10% OS	70% OS, 75% decrease TV	(100)
		EMT-6	–	75% decrease TV	(100)
	Anti-TIM3	CT26	0 and 0% CR	50% CR	(104)
		GL261	? and 0% OS	60% OS	(105)
	Anti-VISTA	CT26	37.5 and 12.5% OS	100% OS	(106)
Anti-CTLA-4+ anti-PD-1 (or anti-PD-L1)	–	B16	10 and 25% OS	50% OS	(92)
		B16.SIY	0 and 0% CR	55.5% CR	(103)
		K7M2	0 and 0% OS	60% OS	(93)
	IDOi (1MT)	GL261	90 and 20% OS	100% OS	(107)
		B16.SIY	55.5 and 0% CR	55.5% CR	(103)

*Note that the response data are not meant for direct comparison among the various preclinical models, but are merely presented to give an overview*.

*CR, complete respone; OS, overall survival; TV, tumor volume; TG, tumor growth*.

*The question mark indicates that the effect of anti-PD-1 was not assessed in this study*.

Furthermore, the inhibitory ligand V-domain immunoglobulin (Ig)-containing suppressor of T cell activation [VISTA, also known as PD-1 homolog (PD-1H)] is expressed on hematopoietic cells (e.g., T cells) and in particular on myeloid cells in tumors (108–110). The combination of anti-VISTA and anti-PD-1 synergistically promoted regression and long-term survival in a colon carcinoma (CT26) model (106). Therefore, it would be interesting to test this combination in the clinic.

In addition to T cell immune checkpoints, NK cells express killer-cell immunoglobulin-like receptors (KIRs), which inhibit the cytotoxic activity of NK cells after interaction with MHC-I on tumor cells (111). Co-blockade of PD-1 or CTLA-4 and KIR might be beneficial due to the activation of both T- and NK cells. Currently, the effect of combination therapy with anti-KIR (lirilumab) and anti-PD-1 (nivolumab; NCT01714739) or anti-CTLA-4 (ipilimumab; NCT01750580) are investigated in phase I clinical studies in patients with advanced solid tumors. In summary, combination therapies with novel immune checkpoints might have a more favorable safety profile compared to anti-CTLA-4 with anti-PD-1, and clinical assessment of these approaches is needed.

### Co-Stimulatory Molecules

Like immune-checkpoint blockade, agonistic antibodies for co-stimulatory molecules (e.g., CD27, CD137, GITR, and OX40) amplify T cell activation and, as a consequence, they may also enhance antitumor T cell responses. Various combinations with checkpoint blockade have been tested in mouse models (Table 2). The combination of OX40 and anti-CTLA-4 is synergistic in ovarian carcinoma (ID8), fibrosarcoma (MCA-205), and prostate cancer (TRAMP1) models (112, 113). Moreover, anti-GITR and anti-PD-1 have synergistic effects in the ID8 model (114). Some of these combinations have potentially less autoimmune side effects than double immune-checkpoint blockade while still having beneficial effects, for example, combination therapy involving anti-CD137 and CTLA-4 blockade in MC38 colon carcinoma tumors and GL261 glioblastoma (115, 116). In contrast, this combination treatment had no effect on B16 melanoma tumors (115, 116), which may be due to increased PD-L1 expression on those tumors.

**Table 2:** **Preclinical studies immune-checkpoint blockade plus co-stimulation**.

Combination		Tumor model	Response monotherapy	Response combination therapy	Reference
Anti-CTLA-4	CD137	CL261	0 and 0% OS	18% OS	(116)
		ID8	No effect	No effect	(117)
		MC38	14 and 14% CR	86% CR	(115)
		B16F1/F10	No effect	No effect	(115, 118)
	OX40	ID8	0 and 0% OS	60% OS	(112)
		MCA-205	18 and 22% OS	75% OS	(113)
		TRAMP-C1	20 and 18% OS	75% OS	(113)
	CD27	TC-1	0 and 50% OS	50% OS	(119)
Anti-PD-1	CD137	B16F10	No effect	85% decrease TG	(120)
		CT26	0 and – CR	100% CR	(121)
		ID8	No effect	Median survival + 30 days	(117)
	GITR	ID8	0 and 0% OS	20% OS	(114)
	CD27	TC-1	0 and 50% OS	100% OS	(119)

*Note that the response data are not meant for direct comparison among the various preclinical models, but are merely presented to give an overview*.

*CR, complete respone; OS, overall survival; TV, tumor volume; TG, tumor growth*.

Since PD-L1 expression can confer resistance to anti-CD137 treatment (122), combined CD137 activation and PD-1 rather than CTLA-4 blockade may further improve antitumor responses. Indeed, anti-CD137 plus anti-PD-1 resulted in increased efficacy in mouse models for colon carcinoma (ID8), melanoma (B16F10), and ovarian carcinoma (ID8) (117, 120, 121). Furthermore, triple therapy (activation of CD137 and blockade of PD-1 and CTLA-4) was even more effective in the ID8 model (117). However, CD137 agonists have been reported to induce severe liver inflammation (123, 124), and, therefore, more studies determining the toxic effects of combinations involving such agonists are needed. Moreover, intratumoral administration may help to minimize liver toxicity. Although liver inflammation was not detected in combination with anti-CTLA-4 or anti-PD-1 in preclinical studies (115, 117, 120), caution should be taken during its clinical assessment. Currently, a phase I study, in which the toxicity of anti-CD137 and anti-PD-1 is determined in patients with solid tumors, is in progress (NCT02179918). Combination approaches with anti-CD27 (Tnfrsf7) are also promising (125). The combination of anti-CD27 with anti-PD-1, but not anti-CTLA-4, eradicated tumors in a cervical cancer model (TC-1) (119). Currently, the combination of anti-CD27 (varlilumab) with anti-CTLA-4 (ipilimumab) or anti-PD-1 (nivolumab) is investigated in respectively patients with melanoma (NCT02413827) and solid tumors (NCT02335918) in phase I/II studies. Like the combination approaches with blockade of novel immune checkpoints, agonists for co-stimulatory molecules might have a more favorable safety profile, but caution is required with anti-CD137.

Summarizing the combinations with T cell stimulation, double immune-checkpoint blockade of CTLA-4 and PD-1 dramatically increased the response rate compared with monotherapy, but personalized treatment of anti-PD-1 with new immune-checkpoint inhibitors or agonists for co-stimulatory molecules can potentially minimize the severe side effects observed with CTLA-4/PD-1 combined blockade.

## Combinations with Trafficking Inhibition of Tregs or MSDCs (Step 4)

Apart from recruiting antitumor T cells in step 4 of the cancer-immunity cycle, tumors also attract MDSCs and Tregs, which contribute to evasion of immune destruction. Therefore, trafficking inhibition of MDSCs and Tregs to the tumor using specific chemokine receptor inhibitors could abrogate immune evasion and improve antitumor T cell responses. Subpopulations of MDSCs express high levels of C–C chemokine receptor 1 (CCR1) or CXC chemokine receptor 2 (CXCR2) (126, 127). Their ligands, respectively, CCL5 (MCP-1), CCL7 (RANTES), and CXCL8, are secreted by tumors and mediate the recruitment of CCR1 or CXCR2 positive MDSCs. Combination of a CCR1 antagonist (CCX9588) and anti-PD-L1 synergistically reduced the tumor burden in a preclinical breast cancer model (126). Moreover, anti-CXCR2 plus anti-PD-1 enhanced survival in a rhabdomyosarcoma (RMS) model (127).

Regulatory T cells (FOXP3^hi^ and CD45RA^−^) express high levels of CCR4 in the blood and inside tumors (128). Therefore, combination approaches of immune-checkpoint blockade and inhibition of Treg recruitment employing anti-CCR4 is promising. In addition, anti-CCR4 promotes antibody-dependent cell-mediated cytotoxicity (ADCC), which may further reduce the Treg population (129). Currently, anti-CCR4 (mogamulizumab) in combination with anti-PD-1 (nivolumab; NCT02705105, phase I/II), anti-PD-L1 (durvalumab; NCT02301130, phase I), and anti-CTLA-4 (tremelimumab; NCT02301130, phase I) is being tested in the clinic in patients with various advanced solid tumors. In summary, preclinical studies indicate the potential of trafficking inhibition of Tregs or MDSCs, but clinical efficacy data are not reported yet.

## Combinations with T Cell Infiltration Stimulators (Step 5)

Deficient T cell infiltration can contribute to tumor immune escape because of the absence of sufficient numbers of T cells. T cell infiltration can be stimulated using anti-VEGF, agonists for innate immune receptors, and epigenetic modification.

### Vascular Endothelial Growth Factor

The growth factor VEGF inhibits T cell infiltration into the tumor by downregulation of adhesion molecules on endothelial cells (130). However, the immunosuppressive function of VEGF is much more diverse. For instance, VEGF also inhibits antigen presentation by DCs, enhances the expansion of Tregs, and mediates PD-1 upregulation on tumor-infiltrated T cells (131–133). High VEGF levels are associated with decreased OS in advanced melanoma patients after treatment with anti-CTLA-4 (134). Therefore, the combination of anti-VEGF-A (bevacizumab; humanized IgG1, Roche) and anti-CTLA-4 (ipilimumab) was investigated in a phase I trial in advanced melanoma patients. This combination resulted in a high CD8^+^ T cell infiltration and a median survival of 25.1 months (135), suggesting a substantial benefit compared to the 10.1 months median survival of anti-CTLA4 alone (9) (note though that this is another trial). In this trial, 28.3% of the patients experienced severe (grade 3 or 4) adverse events (135).

Another phase I trial applying a small molecule tyrosine kinase VEGF receptor (VEGFR) inhibitor (sunitinib) plus anti-CTLA-4 (tremelimumab) to patients with metastatic RCC resulted in acute renal failure, and the study was therefore halted (136). However, combined treatment with the generally safer anti-PD-1 and the VEGFR inhibitor sunitinib may be feasible. In preclinical studies, the combination of anti-VEGF-A and anti-PD-1 therapy was more effective than the monotherapies in a CT26 carcinoma mouse model (131). Moreover, preliminary clinical data of a phase I trial investigating the combination of anti-PD-1 (nivolumab) with either the VEGFR inhibitor sunitinib or with a multikinase VEGFR inhibitor (pazopanib) are promising (137, 138). In summary, combination therapies with anti-VEGF might provide a substantial survival benefit to patients, whereas caution is required with VEGFR inhibitor combination therapies due to renal toxicity.

### Innate Immune Receptors

Innate immune receptors (also known as pattern recognition receptors; PRR) such as TLR can detect a broad range of pathogen- and danger-associated patterns. Binding of these ligands to TLRs on DCs results in their maturation (139, 140). In addition, TLR9 agonists have been shown to increase T cell infiltration in the CT26 colon cancer model (141). Therefore, TLR9 agonists might be used to enhance T cell infiltration into the tumor. Currently, anti-CTLA-4 (ipilimumab) combined with a TLR9 agonist (MGN1703) is studied in a phase I trial in patients with advanced solid malignancies (NCT02668770). In summary, although clinical data are not yet available, combinations with innate immune receptor stimulation might improve T cell infiltration.

### Epigenetic Modulation

Epigenetic silencing of immune-related genes using hypermethylation contributes to immune evasion during tumor progression (142, 143). Hypomethylating agents, of which the nucleoside DNA methylation inhibitor Azacitidine (AZA) and 5-aza-2′-deoxycitidine (5AZA2) are most extensively studied, can be used to restore gene expression (144). 5AZA2 treatment has been shown to promote CD8^+^ T cell infiltration into the tumor in the EL4 lymphoma model, which was attributed to demethylation-induced CD80 expression on tumor cells (145). Therefore, hypomethylation can be used as a strategy to stimulate T cell infiltration into the tumor. Moreover, 5AZA2 treatment combined with anti-CTLA-4 is synergistic in murine mammary carcinoma TS/A and in mesothelioma AB1 models and results in high CD8^+^ and CD4^+^ T cell infiltration (146). Thus, clinical studies are needed as a next step to investigate the safety and efficacy of combination approaches with epigenetic modulation.

In conclusion, several approaches to stimulate T cell infiltration have been attempted but our knowledge on the efficacy of T cell infiltration stimulators combined with checkpoint blockade remains limited. This clearly is an area where more research is needed.

## Combinations with Antigen Recognition Stimulators (Step 6)

Adoptive cell transfer is another promising strategy for the treatment of melanoma with objective response rates of approximately 50% and complete tumor regression in 22% of the patients (147). This approach involves the *ex vivo* culture of a tumor biopsy, the expansion and selection of TILs for tumor specificity, and reinfusion of the tumor-reactive cells into the patient. Instead of TILs, T cells expressing antigen-specific chimeric antigen receptors (CARs) are alternatively used to target specific tumor antigens (148). However, the lack of sufficient T cell infiltration into the tumor, as well as an immunosuppressive tumor-microenvironment may limit ACT success in some patients (149). Indeed, ACT plus anti-PD-1, anti-PD-L1, or anti-CTLA4 synergistically reduced tumor growth in the MC38 carcinoma, B16 and B16F10 melanoma mouse models (150–152) and increased the long-term survival in transgenic Her-2 mice upon ACT of Her-2^+^-specific CAR T cells and anti-PD-1 compared to the monotherapies (153). Combination therapy enhanced the proliferation of T cells within tumors, their cytotoxic activity, and IFN-γ production, which mediated chemokine upregulation (e.g., CXCL10) and further T cell infiltration (150). Thus, the application of immune-checkpoint blockade after ACT may result in complete tumor regression in a large population in the clinic. Currently, the combination of ACT and anti-CTLA-4 is studied in a phase II study in patients with metastatic melanoma (NCT02027935).

Because anti-CTLA-4 and anti-PD-1 promote intratumoral T cell infiltration, immune-checkpoint blockade prior to ACT may increase the number of TILs that can be derived from a tumor biopsy. Moreover, expanded TILs derived from anti-CTLA4-treated patients have a less exhausted phenotype, which is associated with improved clinical responses (154). In summary, applying immune-checkpoint blockade either before or after ACT is a promising approach, but clinical data are currently lacking.

## Combinations That Increase Tumor Killing (Step 7)

As discussed, tumor cells can evade killing *via* PD-L1 expression, since the interaction of PD-L1 with PD-1 inhibits the secretion of cytotoxic mediators by CD8^+^ T cells (2). Moreover, other molecules expressed on APCs or tumor cells may limit killing, such as indoleamine 2,3-dioxygenase (IDO), or immune suppressive mediators accumulating in the tumor environment, like adenosine and transforming growth factor β (TGF-β). Note that also the earlier discussed approaches of chemo- and radiotherapy can help to sensitize tumor cells to T cell-induced death.

### Adenosine

Adenosine inhibits T cell proliferation and cytotoxicity upon binding to the A2A receptor on T cells (155–157). In addition, adenosine can directly provoke metastasis upon interaction with the A2B receptor on tumor cells (158). As a consequence, the combination of an A2A receptor antagonist and anti-CTLA-4 or anti-PD-1 synergistically inhibited (metastatic) tumor growth in breast cancer (4T1) and melanoma (B16F10) preclinical models (159–161). Currently, the safety and tolerability of combined A2A receptor antagonist (CPI-444) and anti-PD-L1 (atezolizumab) treatment is studied in a phase I trial in advanced cancers (NCT02655822).

Adenosine is formed by dephosphorylation of adenosine monophosphate (AMP) by the ecto-enzyme CD73 (162). Thus, CD73 has an immunosuppressive and pro-metastatic effect. Moreover, CD73 stimulates angiogenesis through upregulated VEGF secretion by tumor cells (163). As a consequence, high expression of the enzyme CD73 is associated with a poor prognosis in various cancer types (164–166). This enzyme is also a potential biomarker for anti-PD-1 therapy, since CD73 expression limits anti-PD-1 efficacy (161, 167). Indeed, both combined treatment with anti-CD73 and anti-CTLA-4 or anti-PD-1 enhanced the antitumor activity in colon (MC38), prostate (RM-1), and metastatic breast cancer (4T1.2) models (168). Currently, the combination of anti-CD73 (MEDI9447) plus anti-PD-L1 (durvalumab) is investigated in the clinic in patients with advanced solid cancer (NCT02503774) in a phase I study. In summary, combination approaches with A2A receptor antagonists and anti-CD73 to improve tumor killing are currently studied in the clinic due to their success in preclinical studies.

### Transforming Growth Factor-β

Transforming growth factor-β can also contribute to immune suppression by stimulating Tregs (169). Preclinically, combined treatment with a TFG-β receptor kinase inhibitor I and anti-CTLA-4 synergistically inhibited primary and metastatic tumor growth in a melanoma model (BRAF^V600E^PTEN^−/−^) (170). The combination of the TFG-β receptor kinase inhibitor I (galunisertib) and anti-PD-L1 (durvalumab) or anti-PD-1 (nivolumab) are currently being clinically tested in patients with metastatic pancreatic cancer (NCT02734160, phase I) and NSCLC, hepatocellular carcinoma, or glioblastoma (NCT02423343, phase I/II). These clinical studies will demonstrate if combination approaches with TGF-β receptor kinase inhibitors may improve the treatment of various tumors.

In addition, TGF-β may impair radiotherapy-induced T cell priming, and TGF-β blocking antibodies can enhance T cell priming (step 2–3) and promote abscopal responses induced by (fractionated) radiotherapy in the 4T1 mouse model (53).

### IDO

The presence of the tryptophan degrading enzyme IDO on APCs or tumor cells may also limit the killing efficacy of CTLA-4 or PD-1 blockade because tryptophan is required for T cell proliferation (171). Indeed, several preclinical studies demonstrated a survival benefit of combinations of anti-CTLA-4 or anti-PD-L1 with IDO inhibition by 1-methyl-d-tryptophan (1MT) treatment (Table 1). Thus, combination approaches with IDO inhibition may be efficacious, especially in patients with a high IDO expression. Finally, triple immune-checkpoint blockade of CTLA-4, PD-1, and IDO could further improve antitumor responses, but this approach probably does not increase the effectiveness compared to double checkpoint blockade in more established tumors (107).

Early clinical safety and efficacy data for the IDO1 inhibitor (epacadostat) in combination with anti-PD-1 (pembrolizumab) were promising for the treatment of various advanced cancers in a phase I/II study (e.g., melanoma, RCC, and NSCLC) (172). Currently, the IDO inhibitor epacadostat is also being tested in combination with anti-PD-1 (nivolumab; NCT02327078) and anti-PD-L1 (durvalumab; NCT02318277) for various cancers in phase I/II studies. In addition, 1MT (indoximod) plus anti-CTLA-4 (ipilimumab) or anti-PD-1 is being tested in melanoma patients (NCT02073123) in a phase I/II study. Thus, both preclinical and early clinical studies involving IDO inhibition have yielded promising results. However, because IDO inhibition seems particularly effective in tumors with a high IDO expression, it would be useful to target these patients specifically.

Summarizing the approaches that stimulate killing by T cells, various clinical trials currently investigate combination therapies that enhance such killing, but to date clinical safety and efficacy data are limited.

## Conclusion

Various combination approaches with immune-checkpoint blockade have been studied in preclinical models, but to date the clinical efficacy and toxicity data are limited. It is difficult to compare the efficacy of the preclinical studies, since various cell lines and tumor models are used. A step forward here would be to employ a combination of experimental approaches and mathematical modeling to quantify the CTL-mediated killing rates (173, 174) for tumor destruction in different settings (i.e., tumor models, treatments) as well as the variability of these rates among mice, taking into account factors such as T cell infiltration and tumor size.

Double immune-checkpoint blockade or combinations with agonists for co-stimulatory molecules are effective at amplifying T cell activation. High response rates have especially been obtained by the combination of CTLA-4 and PD-1 and as a consequence this combination was approved in 2015 for the treatment of melanoma. Nevertheless, these approaches are not effective in all patients. Preclinical data suggest that complete tumor regression may be achieved in a large part of the population after combined treatment with ACT and immune-checkpoint blockade, but its widespread employment may be limited due to extensive laboratory requirements and the high costs involved. Initial clinical data also indicate that combination approaches with multi-peptide vaccines are effective, but patients with non-melanoma tumors expressing low levels of tumor antigens do not benefit from such an approach. In order to improve the efficacy of immune-checkpoint blockade in patients with non-melanoma tumors, combinations with chemotherapy or targeted therapies may be effective, since they induce antigen release and provide danger signals, but the adverse effects are often severe. Combined double immune-checkpoint blockade and radiotherapy might be a promising combination therapy in case the abscopal responses can be optimized (Figure 1). Moreover, the optimal timing of immune-checkpoint blockade relative to the “backbone” radiotherapy needs to be determined. Although combination approaches can increase the fraction of patients that respond to treatment in various cancer types, individualized immune-checkpoint combination approaches, based on predictive biomarkers, has an even higher potential to ensure that each patient is provided with the optimal care. Therefore, establishing biomarkers specifically for combination therapies involving immune-checkpoint blockade has high priority in future research.

## Author Contributions

MS and JB conceptualized the manuscript; MS drafted the manuscript and composed the figures; and MS, JB, and IV carefully revised the manuscript. All the authors read and approved the final manuscript.

## Conflict of Interest Statement

The authors declare that the research was conducted in the absence of any commercial or financial relationships that could be construed as a potential conflict of interest.

## Abbreviations

1MT, 1-methyl-d-tryptophan; ACT, adoptive cell transfer; APC, antigen-presenting cell; BRAFi, BRAF inhibitor; CARs, chimeric antigen receptors; CD, cluster of differentiation; CR, complete response; CRPC, castration-resistant prostate cancer; CTL, cytotoxic T lymphocyte; CTLA-4, cytotoxic T lymphocyte antigen-4; CXCL, C–X–C motif chemokine ligand; GITR, glucocorticoid-induced TNFR family-related gene; GM-CSF, granulocyte-macrophage colony-stimulating factor; gp100, glycoprotein 100; GVAX, cellular vaccines consisting of irradiated GM-CSF producing tumor cells; HNSCC, head and neck squamous cell carcinoma; HPV, human papillomavirus; ICD, immunogenic cell death; IDO, indoleamine 2,3-dioxygenase; IFN-γ, interferon gamma; KIRs, killer-cell immunoglobulin-like receptors; LAG-3, lymphocyte-activation gene-3; MAPK, mitogen-activated protein kinases; MART-1, protein melan-A; MEKi, mitogen-activated protein kinase kinase inhibitor; MHC, major histocompatibility complex; NCT, national clinical trial; NK cell, natural killer cell; NSCLC, non-small-cell lung carcinoma; NY-ESO-1, cancer-testis antigen; ORR, overall response rate; OS, overall survival; PD-1, programed death-1; PD-L1,2, programed death-ligand 1,2; PFS, progression-free survival; PSA, prostate-specific antigen; PTEN, phosphatase and tensin homolog; RCC, renal cell carcinoma; SLP, synthetic long peptide; TCR, T cell receptor; TG, tumor growth; TIGIT, T cell ITIM domain; TIL, tumor-infiltrating lymphocyte; TIM-3, T cell immunoglobulin and mucin domain-3; TNFR, tumor necrosis factor receptor; Treg, regulatory T cell; TV, tumor volume; VEGF, vascular endothelial growth factor; VEGFR, VEGF receptor.
